# Continuous Multi-Material Additive Manufacturing

**DOI:** 10.34133/research.0889

**Published:** 2025-09-18

**Authors:** Jiawei Sun, Wangjun Xiong, Lidian Zhang, Xuan Guo, Yanlin Song, Lei Wu

**Affiliations:** ^1^ Key Laboratory of Green Printing, Institute of Chemistry, Chinese Academy of Sciences, Beijing 100190, P. R. China.; ^2^ University of Chinese Academy of Sciences, Beijing 100049, P. R. China.; ^3^ China National Petroleum & Chemical Planning Institute, Beijing 100013, P. R. China.; ^4^ CAS Key Laboratory of Bio-inspired Materials and Interfacial Sciences, Technical Institute of Physics and Chemistry, Chinese Academy of Sciences, Beijing 100190, P. R. China.

## Abstract

Slice-based additive manufacturing has been intensively investigated due to its potential in complex 3-dimensional (3D) structure construction across various fields. Current researches focus on curing surface and resin formation regulation to realize continuous printing. However, multi-material construction necessitates vat switching, compromising construction continuity. Achieving simultaneous multi-material construction within a single layer and enabling continuous multi-material construction continue to pose substantial challenges. Here, we present a continuous multi-material additive manufacturing (CMAM) approach by integrating extruding multi-liquid phases into droplet-based 3D printing system. The multi-droplet-based multi-liquid reservoir enables both 2D patterning of multi-liquid materials and their real-time curing, along with continuous resin replenishment to achieve continuous multi-material 3D construction. Additionally, extrusion parameters (extrusion number, spatial distribution, and extrusion flow rates) are controllable layer by layer, leading to controllable muti-material 3D distribution. Interfacial fusion can be controlled by adjusting printing speed and resin viscosity, leading to enhanced mechanical adhesions of 2 materials without influencing interfacial boundary precision. Increasing extrusion number can realize multi-material 3D structure construction with controlled material distribution, which can be extended to 3D structure-based anti-counterfeiting and soft robotics, guaranteeing a highly efficient and sustainable approach to multi-material 3D fabrication.

## Introduction

Additive manufacturing, which is also well known as 3-dimensional (3D) printing, is a sustainable fabrication approach that has evolved into a powerful platform for 3D structure construction due to its design freedom, ease of operation, and mold-free processing [1–3]. Its applications span structural materials [4–7], sensors, devices [8–12], soft robotics [13–17], and tissue engineering [18–22]. Notably, among various 3D printing techniques, digital light processing stands out by projecting full-layer ultraviolet (UV) patterns, which enables high-speed production for instantaneous photopolymerization. Since the voxel-to-slice conversion step is skipped, the manufacturing process becomes faster [23–26]. A typical 3D printing setup consists of 3 primary components: (a) a UV light source that projects sliced layer patterns, (b) a resin container serving both as a reservoir for liquid photopolymer and as the curing surface for UV-induced polymerization, and (c) a moving platform with an attached supporting plate where the 3D structure is progressively fabricated. The UV polymerization process of liquid resin at the curing surface to form cured structure is the basic process to prepare one slice. Recent studies have demonstrated that optimizing the fabrication process through interfacial engineering can improve both manufacturing efficiency and product quality [27]. Specifically, the introduction of a dynamic, liquid-like separation layer between the curing interface and printed structure minimizes interfacial adhesion forces, facilitating continuous fabrication while maintaining dimensional accuracy [28,29]. The inert “liquid-like” separation layer can be realized through various approaches based on curing surface modification, including the “dead zone” mechanism in the continuous liquid interface production technology [30], low-adhesive “slippery curing surfaces” [31], and the “flowing immiscible fluorinated oil with cooling systems” in the high-area rapid printing technology [32], or advanced resin formulations [33–35] employing multiple photoinitiators matched with corresponding multiple UV light. Beyond conventional interface regulation, construction control can be attained by increasing or decreasing the involved interfaces. The establishment of a 3-phase contact line (TCL) [36] facilitates droplet-based manufacturing with superior material utilization, achieved through precise manipulation of the receding TCL. Rotational slicing about the symmetric axis (versus conventional *z*-axis motion) enables curing surface-independent volumetric manufacturing. Furthermore, integrating extra microfluidic [37], electric [38,39], and magnetic [40] control systems at curing interfaces allows precise modulation of resin properties and material composition during printing.

However, there exists a fundamental trade-off between construction continuity and simultaneous multi-material deposition. State-of-the-art multi-material 3D printing employs sequential resin exchange in the printing vat to change the resin employed for the current layer [41–46]. The vat switching procedure necessitates interruption of ongoing layer polymerization, thereby preventing continuous multi-material deposition within individual layers [47,48]. While deposition-based techniques like direct ink writing (DIW) [49,50] and inkjet 3D printing [51] can achieve continuous multi-material fabrication through multi-nozzle systems with dynamic material switching of nozzles [52,53], their voxel-by-voxel deposition mechanism necessitates sequential *x*–*y* plane patterning followed by *z*-axis stacking. These approaches result in comparatively slower build rates and reduced resolution relative to photopolymerization methods (Table S1), making continuous slice-based multi-material polymerization an unresolved challenge.

Herein, we present CMAM approach that utilizes patterned slices as the fundamental molding unit. Drawing inspiration from the on-demand fabrication capabilities of extrusion-based 3D printing technique [54] and one-droplet 3D printing methodology [55], we introduce controlled number and distribution of extrusion devices at the curing surface. Based on the retractable TCL endowed by the droplet reservoir, multi-liquid can be patterned inside the reservoir through continuous extrusion. With continuous UV curing depleting the liquid resin extrusion, droplet-based 3D printing enables simultaneous multi-material extrusion and curing in a continuous process. By precisely controlling resin extrusion parameters (rate, volume, and spatial distribution), this approach can fabricate controllable planar-to-3D interfaces with 4 or more materials simultaneously in a single layer and finally enables a continuous multi-material construction process. With liquid phase contact and simultaneous UV polymerization, interfacial mechanical performance was enhanced through interfacial fusion while maintaining sharply defined 3D structural boundaries. Leveraging precise control over material composition and spatial distribution, this method enables single-layer-based multi-material fabrication and further the complex multi-material structures. These include 3D anti-counterfeiting features with customized material patterns and magnetic-responsive fish constructs with programmed movement trajectories. The system’s advantages of uninterrupted multi-material construction and scalable droplet-based build volume expand the range of achievable materials, geometries, and practical applications in additive manufacturing.

## Results

### CMAM conception and configuration

**Fig. 1.**
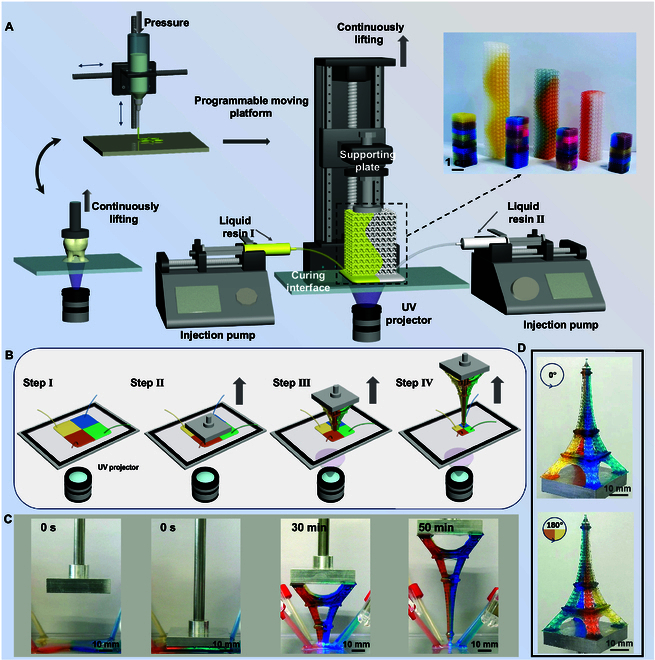
Schematic configuration of CMAM system. (A) Schematic demonstration of the CMAM system. (B and C) Scheme (B) and time-lapse (C) images of the 4-material Eiffel Tower structure construction process by the CMAM approach. (D) Multi-angle optical microscopy images of the fabricated Eiffel Tower microstructure. (E) Optical images of the examples of multi-material 3D structures printed by this strategy.

Figure 1A shows the schematic CMAM system, consisting of 2 key components: (a) a bottom-up continuous manufacturing module for instantaneous slice curing, stacking, and structure fabrication [31], and (b) an extrusion-based resin delivery system that maintains droplet-based material reservoirs while providing real-time resin replenishment. Notably, the self-built bottom-up apparatus features a programmable motion stage with a supporting plate, a UV-transparent curing interface, and a digital UV projector. Scaling to larger construction areas can be accomplished either by deploying multiple *x*–*y* plane UV projectors or by scanning a single projector, which will compromise the printing continuity. The extrusion device is also self-built, which is composed of a disposable infusion tube attached through a syringe mounting on a pump. The resin delivery tubes should be positioned exclusively at the TCL, taking care to remain outside the UV projection zone. This placement strategy prevents tube curing while maintaining consistent material delivery during the printing process.

The process begins with the precise deposition of multi-material patterned droplet onto the curing surface, achieved through controlled extrusion parameters (extrusion number, spatial distribution, and extrusion flow rates) that faithfully reproduce the digital spatial material design (step I). Then, the supporting plate moves downward to contact the curing surface with certain thickness of liquid resin in step II. Then, patterned layers are UV cured as the support plate ascends, synchronized with the droplet reservoir’s TCL receding (step III) [56]. By continuously replenishing diverse liquid resins into the droplet reservoir according to design, 3D structure with multi-material 3D distribution can be printed with high structural precision, stability, and material efficiency (step IV).

Figure 1B demonstrates the continuous printing process of an Eiffel Tower with 4 different colors, achieved using 4-side extrusion resin delivery tubes operating at identical, real-time-adjusted extrusion rates as Fig. S1 that vary with slice area, resulting in 4 linear interfaces and uniform color distribution (Fig. 1C). The resin delivery tubes should be positioned exclusively at the TCL when the printed patterns become smaller. The distribution of the 4 used materials is confirmed by the optical images captured from multiple capturing angles listed in Fig. 1D. In addition, by regulating corresponding extrusion parameters (extrusion number, spatial distribution, and extrusion flow rates), CMAM with sharp interfacial boundaries is capable, allowing the creation of structurally identical objects with varying internal material arrangements. In detail, it is capable to print 2-material gyroid structures featuring both planar and curved interfaces, and multi-material gyroid structures with controlled material distributions in both the *x*–*y* plane and *z* direction, as shown in Fig. 1E, representing a substantial advancement in additive manufacturing’s design space and functional capabilities.

### Continuous bimaterial construction

**Fig. 2.**
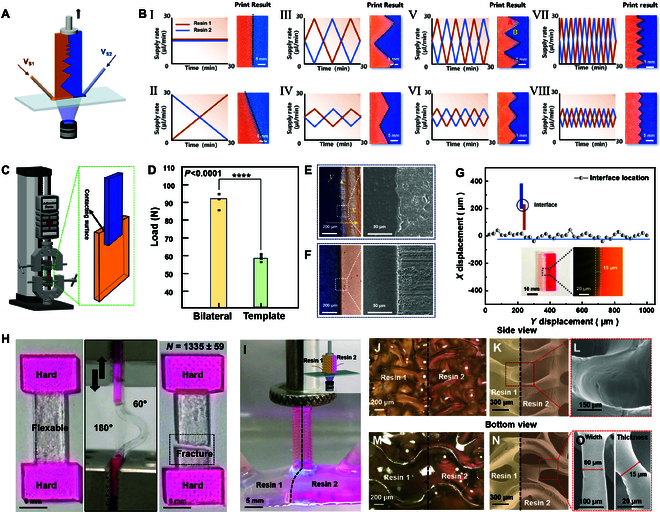
Bilaterally extruded printing of 2 materials and interfacial analysis. (A) Fabrication scheme for 2-material lamellar structures. (B) Time-dependent extrusion rate modulation and resulting 2-material lamellar structures produced at 20 μm/s supporting plate lifting rate. Eight characteristic interface geometries (I to VIII) illustrate the programmable control range. Representative examples (groups I to VIII) demonstrate the range of achievable architectures. (C) Test scheme for interfacial mechanical performance of bimaterial samples. Blue part is commercial Castable Wax 40 Resin, Formlabs; orange part is commercial Clear Resin, Formlabs dyed orange. (D) Statistical significance of the fracture force extracted of samples prepared from bilateral extrusion and template replication approach. (E) Optical and SEM characterization of the sample interface prepared by bilateral extrusion approach. (F) Optical and SEM characterization of the sample interface prepared by template replication approach. (G) Microstructural interface profile observed in the *x*–*y* plane of (E) the bilateral extrusion-printed lamellar structure. Insets show schematic and fluorescence micrographs of the interface formed between Clear and Elastic resins via bilateral extrusion. (H) Long-term cyclic mechanical characterization of the Hard–Flexible–Hard structure (pink: Hard, Clear with pink dye; transparent: Flexible, Elastic). (I) Optical capture of the downsized 2-material gyroid structure construction configuration; inset is the corresponding scheme. (J) Microscopic image of the area around side interface. (K) SEM image of the area around side interface. (L) SEM image of the enlarged part of (K). (M) Microscopic image of the area around bottom interface. (N) SEM image of the area around bottom interface. (O) SEM image of the enlarged part of (N) from the side view (left) and the top view (right).

The capability and property of continuous construction of 2 materials are first investigated [57–61]. Figure 2A presents the bilateral extrusion method for creating lamellar structures featuring programmable linear and curved zigzag interfaces. Two commercial liquid resins, which are dyed blue and orange, are extruded simultaneously in the experiment. Their extrusion flow rates are dynamically adjusted timely, ensuring that the combined extrusion rate matches the resin consumption required for layer curing. When equal extrusion rates are maintained, this process yields lamellar structures with perfectly vertical interfaces, as displayed in Fig. 2B (I). By systematically varying the relative extrusion flow rates over time, interface morphology can be precisely controlled, producing tilted linear interfaces (Fig. 2B, II) and zigzag patterns with tunable amplitude and frequency (Fig. 2B, III to VII, and Movie S1). As detailed in Fig. 2B (I to VIII), the left panels display time-dependent extrusion profiles for each material, with orange and blue curves corresponding to the respective dyed resins (quantitative parameters provided in Table S2). Optical images (right) show printed lamellar structures with controlled linear, tilted, and zigzag interfaces, fabricated using the extrusion profiles illustrated at the left. Using Fig. 2B (V) as an example, the initial extrusion rates of the orange and blue resins are equal (15 μl/min), positioning the interface at the center of the lamellar structure. As the supporting plate continuously ascends, the extrusion rate of the orange resin linearly increased to 30 μl/min, while that of the blue resin decreased to 0 μl/min over 2.5 min, forming interface A (right side of Fig. 2B, V). Subsequently, the orange resin’s extrusion rate linearly dropped to 0 μl/min and the blue resin’s rate rise to 30 μl/min over 5 min, producing interface B (right side of Fig. 2B, V). By further following the regulation curve (left side of Fig. 2B, I to VIII), various zigzag interfaces can be fabricated. This demonstrates that CMAM system enables precise spatiotemporal control over material deposition and solidification in planar dimensions. The building slice of 2D multi-material patterned liquid can thus allow simultaneous control of interfacial material distribution and continuous construction.

The interfacial mechanical behavior of multi-material structures produced via bilateral extrusion printing was investigated through tensile tests aligned with the interface, as displayed in Fig. 2C. The test specimens consisted of 2-lamellar structures with defined contact areas (Fig. 2C, inset), where each material’s edges were clamped to the force sensor. A template-replicated sample (Fig. S2) is prepared and served as the control for comparison. During tensile test as shown in Movie S2, the bilaterally extruded samples fractured at the single-material structural side, whereas the control samples failed at the interface. Both the force–displacement profile (Fig. 2D) and fracture force statistics (Fig. S2C) confirm superior interfacial strength in bilaterally extruded samples. Further, optical and scanning electron microscopy (SEM) analyses demonstrate rougher interfacial morphology in bilaterally extruded samples (Fig. 2E) versus template-replicated ones (Fig. 2F). The SEM images profiling of the interfacial transition zone (Fig. 2G) demonstrates that mechanical property enhancement correlates with a ~100-μm-wide fusion zone of 2 materials, where partial polymerization of both resins creates a graded interphase with superior adhesion properties. Then, it is experimentally found that the fusion width is affected by the viscosity difference between the 2 extruded resins. By testing 4 commercial resins including Clear, Grey, Wax, and Elastic (Formlabs, USA) with viscosities of ~1,049, ~1,875, ~2,003, and ~3,914 mPa·s, respectively, 6 distinct 2-material interfaces were successfully printed (Table S3). In single-material extrusion, higher resin viscosity requires greater driving force, typically achieved by increasing the extrusion rate. The observed fusion width is comparable to that of existing discontinuous and continuous multi-material 3D printing approaches [41,49]. Remarkably, the continuous extrusion strategy enhances interfacial adhesion without compromising printing precision (Table S1), overcoming the traditional trade-off between interfacial strength and dimensional fidelity in multi-material construction.

Furthermore, increasing the viscosity difference between the 2 liquid resins results in a narrower fusion width. The smallest achievable fusion width is ~15 μm, observed between Clear and Elastic resins, which have the largest viscosity difference, as shown in the confocal fluorescence image (Fig. 2G, inset). To evaluate long-term interfacial mechanical performance, we designed and printed a Hard–Flexible–Hard structure with interfaces parallel to the curing surface, leveraging interfacial fusion for enhanced mechanical properties. Notably, the fusion width (~100 μm) in this configuration is larger than in vertically oriented interfaces formed with the same materials. This difference likely arises from incomplete consumption of the Hard resin and rapid replenishment of the Flexible resin for ensuring a continuous liquid layer. Cyclic bending tests (0.2 Hz, 60° to 180° range; Fig. 2H and Fig. S3) demonstrate that the structure can withstand 1,335 ± 59 cycles (*n* = 5) before fracturing in the flexible middle section rather than at the interface, confirming robust interfacial durability and long-term mechanical stability of the multi-material extrusion method.

Then, construction stability is evaluated through comparative analysis between conventional single-material vat photopolymerization and the proposed bilateral multi-material extrusion process. In conventional continuous vat photopolymerization, structural adhesion to the curing interface occurs after prolonged fabrication periods, ultimately resulting in print failure (Fig. S4). In contrast, the continuous bilateral extrusion approach enables fabrication of much taller structures without interfacial adhesion or print failure, which is finally limited only by the maximum travel of the moving platform. This capability permits extended printing durations and greater build volumes of the droplet-based printing system. Finally, to determine the system’s lower resolution limits, we performed systematic downscaling of gyroid structures, testing dimensions ranging from the UV projector’s nominal resolution to sub-resolution features. As shown in Fig. 2I, the downsized structure was fabricated by co-extruding 2 liquid resins at identical flow rates, resulting in the formation of a well-defined planar interface. Then, the side and bottom morphologies of the fabricated structures were systematically characterized (Fig. 2J to L and Fig. 2M to O, respectively). The measured structural resolution corresponds with the UV projector’s nominal specifications. Remarkably, features as small as ~15 μm were achieved (Fig. 2O) along the printing direction, which can be attributable to the improved fabrication accuracy and stability afforded by the one-droplet 3D printing strategy [36].

### Interfacial morphology control and multi-material construction

**Fig. 3.**
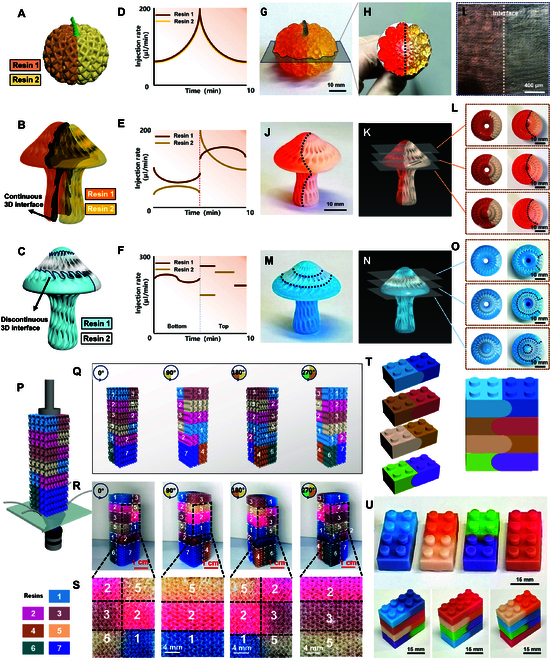
Interfacial morphology control and continuous multi-material construction. (A) Digital 3D representation of the geometry of pumpkin-shaped structure. (B) Digital 3D representation of the geometry of mushroom-shaped structure featuring 3D continuous interface. (C) Digital 3D representation of the geometry of mushroom-shaped structure featuring 3D discontinuous interface. (D) Time-dependent extrusion rate profile for fabricating the pumpkin-shaped structure featuring inner 2D planar interface. (E) Time-dependent extrusion rate profile for fabricating mushroom-shaped structures featuring 3D continuous interfacial. (F) Time-dependent extrusion rate profile for fabricating mushroom-shaped structures featuring 3D discontinuous interface. (G) Optical characterization of the bilaterial extrusion-printed pumpkin-shaped structure. (H) Cross-sectional optical micrograph of the bilaterial extrusion-printed pumpkin-shaped structure. (I) High-magnification optical image of (H). (J) Optical characterization of the bilaterial extrusion-printed mushroom-shaped structure featuring 3D continuous interface. (K) Cross-sectional scheme of mushroom-shaped structure featuring 3D continuous interface. (L) Cross-sectional optical image interfaces in (K). (M) Optical characterization of the bilaterial extrusion-printed mushroom-shaped structure with 3D discontinuous interface. (N) Cross-sectional scheme mushroom-shaped structure featuring 3D discontinuous interface. (O) Cross-sectional optical image interfaces in (N). (P) Schematic diagram of the 7-color multi-material structure 3D representation and its construction configuration. (Q) Schematic diagram of the 7-color multi-material characterized from different directions. (R) Optical images of the printed 7-color multi-material 3D structure characterized from different directions. (S) Enlarged optical images in (R). (T) Schematic models of 4 block structures with different material distributions, and scheme of the stacking result. (U) Optical images of the bilaterial extrusion-printed 4 block structures with different material distributions, and experimental realization of material 3D distribution regulation by stacking the 4 block structures with different sequences.

Building upon the demonstration of 2D multi-material patterned liquid as building slice with uninterrupted fabrication continuity, the extended capability for 3D material distribution control is further explored. Three distinct architectures were computationally designed to validate this approach: porous pumpkin-shaped structure featuring 2D planar interface (Fig. 3A), and 2 mushroom-shaped structures featuring fundamentally different 3D interfacial configurations (Fig. 3B and C). With real-time modulation of phase-specific extrusion parameters (Fig. 3D to F), precise fabrication of pumpkin-shaped structure maintaining perfect 2D interfacial planes (Fig. 3G) and mushroom-shaped structures with either continuous (Fig. 3J) or discontinuous (Fig. 3M) 3D interfaces can be bilateral extrusion printed. Multi-plane cross-sectional characterization (Fig. 3H and I, K and L, and N and O) reveals exceptional fidelity between as-printed and designed material distributions, confirming the reproducibility and spatial resolution of the bilateral extrusion platform.

By enabling simultaneous control over extruded material composition and number, the extension of this strategy in continuous 3D multi-material fabrication and its versatility in 3D control over material distribution and interfacial morphology are further investigated. For instance, by employing multi-delivery tubes and the regulation of extrusion number, distribution, and extruded material sequence, complex gyroid architectures can be realized with bimaterial systems featuring engineered planar and nonplanar interfaces, and multi-material variations with spatially programmed compositions in all 3 dimensions. In addition, Fig. 3P to R demonstrates excellent agreement between the fabricated 7-material structures and their digital models across all viewing angles, with precisely reproduced interfaces and material distributions matching the design specifications (Fig. 3S). Moreover, complex 3D material arrangements can be realized by layering structures featuring 2-material distributed block structures shown in Fig. 3T and U. Diverse 3D material configurations can be achieved by adjusting the stacking sequence, validating the capability to print 3D structures with user-defined material arrangement. As the core of this method employs patterned slices as molding unit, it is limited by the number of material and multi-material distribution complexity in current slice, material continuity during switching of different slices, and the increase or decrease in material species. The single-layer pattern is influenced by the pattern availability of the extrusion tube loading with different materials. Further, the requirement of interface boundary precision also influences the way of material switching and printing continuity. Based on different actual printing need, the extrusion device can be modified or corresponding auxiliary device can be added to further realize more flexible multi-material patterns along the current line.

### Applications of CMAM approach

**Fig. 4.**
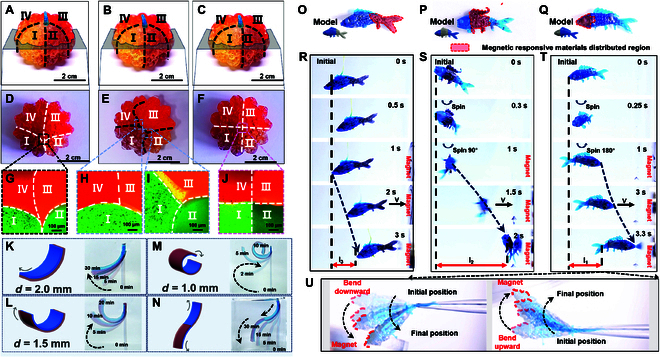
Applications of CMAM approach. (A to F) Multi-material pumpkin-shaped structures exhibiting uniform exteriors with varied internal material arrangements. (A to C) Lateral optical images showing the surface characteristics of the pumpkin-shaped structures comprising 4 materials. (D to F) Cross-sectional optical images showing the internal distributions of the pumpkin-shaped structures comprising 4 materials featuring nonuniform (D and E) and uniformly quaternary (F) distributions. (D) to (F) display the corresponding cross-sectional views in (A) to (C), respectively. (G to J) Fluorescence images showing the internal distributions of the pumpkin-shaped structures comprising 4 materials featuring nonuniform (G) and uniformly quaternary (J) distributions, and the dual 3-phase interfaces within individual structures (H and J). (K to N) Schematic diagram and sequential optical micrographs showing the morphing evolution of multi-material structures fabricated via continuous bilateral extrusion. (K) Schematic diagram and time-lapse images showing the morphing evolution of a 2-mm-thick 2-material laminated structure (equal 1-mm phase thickness; 2 × 16 × 40 mm). (L) Schematic diagram and time-lapse images showing the morphing evolution of a 1.5-mm-thick 2-material laminated structure (equal 0.75-mm phase thickness; 1.5 × 16 × 40 mm). (M) Schematic diagram and time-lapse images showing the morphing evolution of a 1-mm-thick 2-material laminated structure (equal 0.5-mm phase thickness; 1 × 16 × 40 mm). (N) Schematic diagram and time-lapse images showing the morphing evolution of a 2-mm-thick 4-material laminated structure (equal 1-mm phase thickness; 2 × 16 × 40 mm). (O to Q) Scheme and optical characterization of 3D fish structures featuring magnetic-responsive materials at distinct anatomical positions: tail (O), abdomen (P), and head (Q). Schematics in (O) to (Q) illustrate the models of 3D fishes. (R to T) Sequential optical micrographs recording the dynamic behavior of fish constructs with regional magnetic responsiveness: (R) tail distribution, (S) abdomen distribution, and (T) head distribution during magnetic field application. (U) Magnetic field-induced head flexion in the fish featuring magnetic-responsive materials located at the head.

Leveraging precise control over material composition, spatial distribution, and interfacial morphology, we further demonstrate how this strategy enables advanced applications in anti-counterfeiting, shape-morphing structures, and soft robotics, which expands both the design possibilities and practical applications of 3D printing [62]. Extending beyond 2-material fabrication, this method enables single-process printing of 4-material pumpkin structures featuring control over surface exterior and internal composition distribution (Fig. S5 and Movie S3). Controlling the extrusion flow rates of the 4 kinds of materials, it is capable to fabricate multi-material pumpkin-shaped structures with identical exteriors (Fig. 4A to C) but differing internal distributions including nonuniform (Fig. 4D and E) or uniformly quaternary (Fig. 4F) distributions. Fluorescence microscopy clearly delineates all material interfaces, demonstrating both nonuniform (Fig. 4G) and uniform (Fig. 4J) 4-phase junctions, as well as dual 3-phase interfaces within individual structures (Fig. 4H and I). It should be mentioned that the yellow region in Fig. 4I is due to the physical overlap of the structures, rather than fusion of the 2 materials, as displayed in Fig. S6. The nondestructive nature of these 4-material pumpkin structures conceals their internal distribution patterns, making them ideal for 3D printing anti-counterfeiting applications. This characteristic expands 3D printing’s potential for creating complex 3D structures with precisely controlled material arrangements. While the present multi-material 3D printing process terminates upon completion of resin extrusion and UV projection, advancements can be achieved through system integration of machine learning-based optimization of extrusion system [51,63], through which real-time feedback control of extruded materials can be realized with improved system intelligence.

This strategy can also be extended in fabricating shape morphing structures by combining materials with distinct swelling properties, which is helpful for application in soft robotics. Upon immersion in anhydrous ethanol (Fig. S7), the asymmetric swelling behavior of the constituent materials induces curvature toward the component exhibiting lower volumetric expansion (Fig. 4K to N). By tuning the relative thickness of each side or the total thickness (*d*), the bending direction and magnitude are precisely controlled: Thinner structures exhibit faster curling speeds and greater deformation (Fig. 4K to N). Further control over bending morphology is achievable by increasing structural complexity through tailored material distribution and extrusion rates. For example, by designing a lamellar structure with opposing material arrangements in 2 sections, an S-shaped deformation is obtained upon swelling (Fig. 4N). Moreover, the method’s high spatial material distribution control also enables printing of magnetic-responsive structures with programmable motion trajectories (Movie S4). For instance, 3D fish structures featuring regional magnetic responsiveness are bilateral extrusion printed through resins comprising flexible resin and Ni-loaded hard resin. Three fish structures with magnetic-responsive materials localized at the tail (Fig. 4O), abdomen (Fig. 4P), and head (Fig. 4Q) are designed and printed. Under a magnetic field, the tail-responsive fish moves directly toward the magnet (Fig. 4R), while the abdomen- and head-responsive variants first rotate 90° or 180°, respectively, before aligning to the magnet (Fig. 4S and T). Additionally, the head-responsive fish exhibits bending motion along with the magnet (Fig. 4U), highlighting the precision of this approach in governing 3D material arrangements and resultant functional behaviors.

## Conclusion

In summary, this work demonstrates a continuous multi-material additive manufacturing (CMAM) strategy that simultaneously regulates solid–liquid and liquid–liquid interfacial properties. By preserving the droplet reservoir and receding TCL during UV curing, this approach eliminates volumetric construction constraints of one-droplet 3D printing while maintaining high material efficiency. Precise control over extrusion rates, material number, and spatial distribution enables the concurrent printing of 4 or more materials with programmable 2D (planar/curved) or 3D interfacial architectures. Furthermore, the application of fabricating anti-counterfeiting structures with spatially defined material distributions and 3D magnetic-responsive fish exhibiting programmable motion trajectories are realized, which expands the versatility and application scope of 3D printing. The proposed method provides unparalleled control in constructing intricate multi-material architectures, offering a transformative platform for continuous on-demand 3D fabrication of functional devices.

## Materials and Methods

Commercial liquid resins from Formlabs (USA) including Elastic 50A resin, Castable wax 40 resin, Grey Pro resin v1, Clear resin, and Color kit resin (comprising a base resin and 5 color pigments) are employed. Specifically, Fig. 2’s bimaterial structures with adjustable interfaces were printed from blue- and orange-loaded Clear resin. Mechanical test samples are fabricated from Clear resin (orange-dyed) and Castable Wax 40 resin. Multi-material characteristic structures utilized Color Kit resin to produce opaque matte parts in diverse colors. Shape-morphing structures are from Castable Wax 40 resin and red-dyed Elastic 50A resin. Rheology influenced fusion studies employed Elastic 50A resin, Castable wax 40 resin, Grey Pro resin v1, and Clear resin. Hard–Flexible–Hard structures for long-term interfacial mechanical testing were printed using pink-dyed Clear resin and Elastic 50A resin. The used resins in the same slice should be with the same curing volume under the same UV intensity.

### Custom additive manufacturing system

The custom-designed additive manufacturing system comprised a 405-nm light-emitting diode (LED) UV projector (PRO6500S, Wintech, China, with projection area of 150 mm × 84 mm, resolution of 1,920 × 1,080 pixels, and adjustable intensity of 0 to 65 mW/cm^2^), a liquid resin vat featuring a UV-transparent curing interface (quartz cell with perfluorocarbon-swollen polydimethylsiloxane-coated bottom), and an aluminum supporting plate (self-made) mounted on a programmable moving platform (MC600, Zolix Instruments Co. Ltd., China, offering translation speed of 1.5 to 100 mm/min) from bottom to up as displayed in Fig. 1A. The slicing thickness is set as 25 μm. All structures are printed under a printing speed of 3 mm/min.

### Extrusion system

The extrusion setup comprises syringe pumps (LSP02-2B and dLSP520 Pro, Longer, China), injection syringes as resin container, and infusion tubes (1.5 mm diameter and 20 cm length) for resin delivery. The LSP02-2B syringe pump (dual-injection channels, linear velocity covering 5 μm/min to 130 mm/min, 140-mm maximum stroke with resolution of 0.156 μm/μstep) is used for resin extrusion for thermal phenomenon studies. The dLSP520 Pro syringe pump (dual-injection channels, linear velocity covering 0.04 μm/min to 86.4 mm/min, 108-mm maximum stroke with resolution of 0.015625 μm/μstep) is used for programmable resin extrusion in CMAM. During construction, the infusion tube and syringe are wrapped with tin paper to prevent liquid resin from exposure to light.

### Post-processing for additive manufacturing

Following fabrication, absolute ethanol washing was performed until the rinse liquid appears clear to remove the residual ink on the printed structure and supporting plate. The sample is then detached from the support plate and subjected to post-curing in a UV LED chamber.

### Characterization

Thermal imaging is characterized through an infrared camera (FLIR A655sc, USA) for real-time temperature dot or linear analysis. Process visualization is conducted through a digital camera (CU-VF100AC, JVC, Japan), through which optical images are captured for real-time monitoring of the construction process and structural evolution. Interface characterization is characterized using an optical microscope (BX53, OLYMPUS, Japan), scanning fluorescence microscope (FV1000-IX81, Olympus, Japan), and SEM (S-4800, Hitachi, Japan) at an accelerating voltage of 5.0 kV. Mechanical testing is realized through a digital load cell (M5-200, force threshold of 1,000 N, force resolution of 0.1 N, Mark-10 Corporation, USA) mounting on the programmable moving platform (ESM303, displacement resolution: 10.0 μm, Mark-10 Corporation, USA). Real-time force measurements were acquired synchronously with clamp displacement, enabling the construction of force–displacement profiles.

## Data Availability

All relevant data is present in the article and the Supplementary Materials.
