# Supplementary material for: Continuous Multi-Material Additive Manufacturing
Source: Research (Wash D C). 2025 Sep 18;8:0889. doi: 10.34133/research.0889 (PMC13182896; doi:10.34133/research.0889)
Supplement: Supplementary 1 — Figs. S1 to S7 Tables S1 to S3 Movies S1 to S4 [file research.0889.f1.zip › sm.docx]

**Supporting Information**

**Continuous Multi-material Additive Manufacturing**

Jiawei Sun, Wangjun Xiong, Lidian Zhang, Xuan Guo, Yanlin Song, Lei Wu

**Email:**  wulei1989@ iccas.ac.cn; ylsong@iccas.ac.cn

**This PDF file includes:**

Figures S1 to S7

Tables S1 to S3

Movies S1 to S4


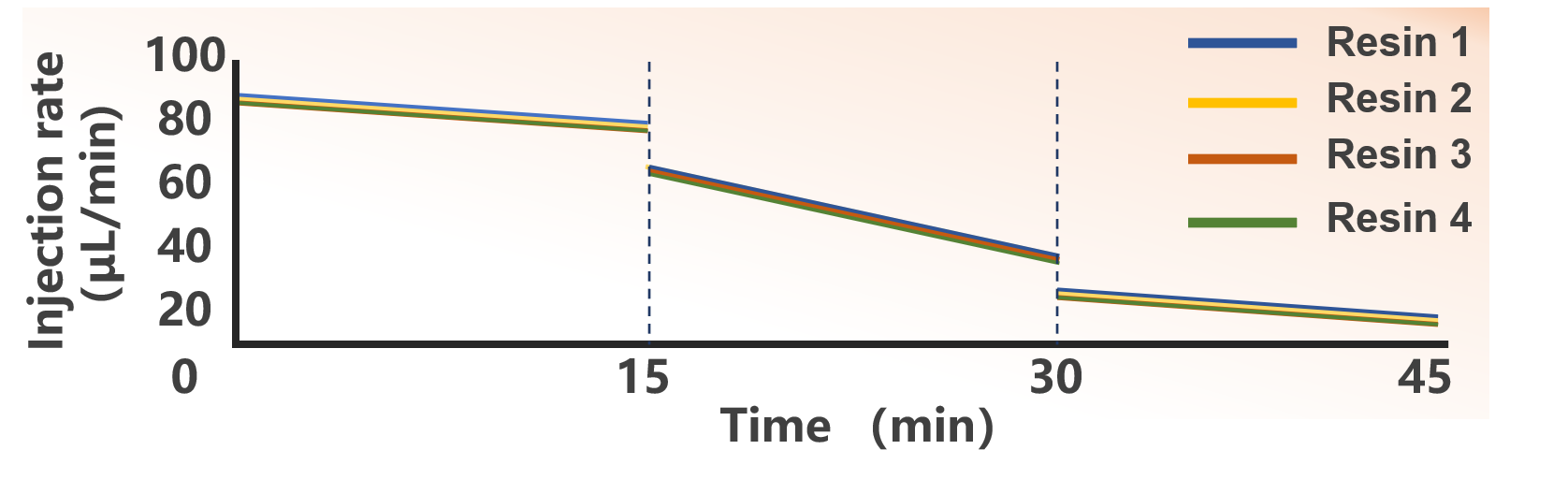


**Fig. S1** Supply rate curves of the four-color Eiffel tower at different printing times.


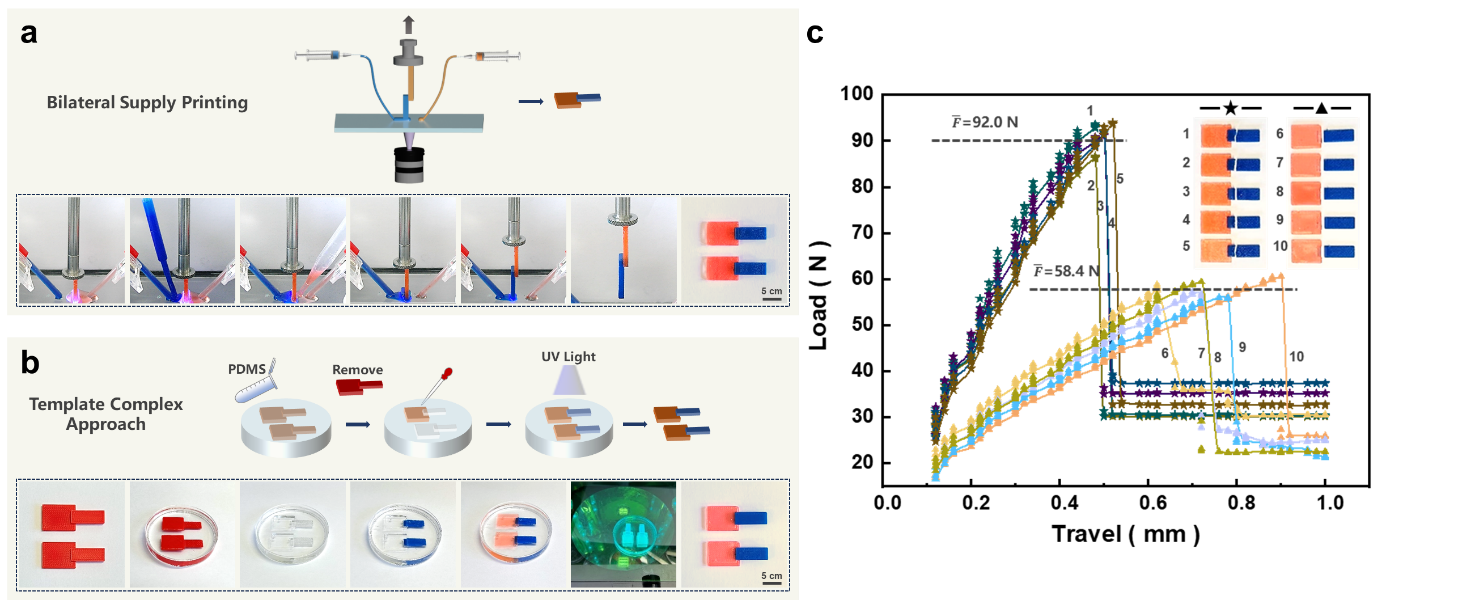


**Fig. S2.** Schematic and time-sequence optical images of the preparation of interfacial mechanical property test samples prepared by bilateral extrusion construction (a) and template replication approach (b). The bilateral extrusion construction method can realize one-step construction while template replication approach needs to prepare the die at first, then solidify the two materials respectively, and finally release the sample, which requires cumbersome construction. (c) Tensile load-displacement curves comparing interface performance of bilaterally extruded samples (triangles, curves Nos. 1-5) and template replication approach (stars, curves Nos. 6-10). Five replicates per condition (n=5) demonstrate reproducibility. The inset shows post-fracture optical micrographs, revealing distinct failure modes between fabrication methods.

**
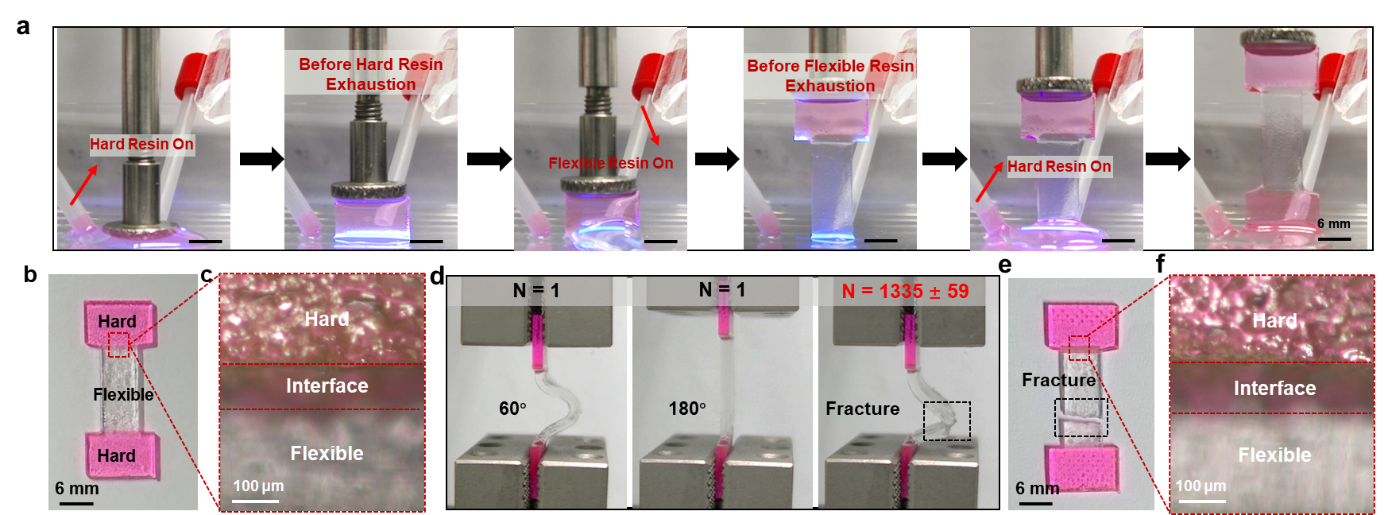
**

**Fig. S3.** Long term mechanical test of Hard-Flexible-Hard structure with interfaces parallel with the curing surface. (a) Optical captures of continuous construction of Hard-Flexible-Hard structure (pink: Hard, Clear with pink dye; transparent: Flexible, Elastic) (b) Optical image of the Hard-Flexible-Hard structure for long-term mechanical testing. (c) Enlarged optical image of the interfaces in (b). (d) Optical captures of cyclic tests at a frequency of 0.2 Hz with sample bending angles ranging from 60° to 180°. (e) Optical image of the sample after fracture at the flexible part. The structure can sustain 1335 ± 59 (n = 5) cycles before fracture at the middle flexible part rather than at the interface. In detail, the extrusion of Hard resin should be quantitative according to the hard structure height and cross-section. Then before the complete exhaustion of Hard resin, Flexible resin is supplied with quantitative volume according to the flexible part volume in a fast extrusion rate. If Flexible resin is supplied when Hard resin is completely exhausted, the construction should be stopped to ensure the next liquid layer supplementation for curing, otherwise defects caused by untimely resin refilling will occur. The switching to the next hard resin is conducted with the same procedure, through which Hard-Flexible-Hard structure is printed. The fusion width is ~ 100 μm, which is larger than the interface vertical to the curing surface formed by the same materials, which can be due to the incomplete consumption of Hard resin and fast supplementation of the Flexible resin. (f) Enlarged optical image of the interfaces in (e).

**
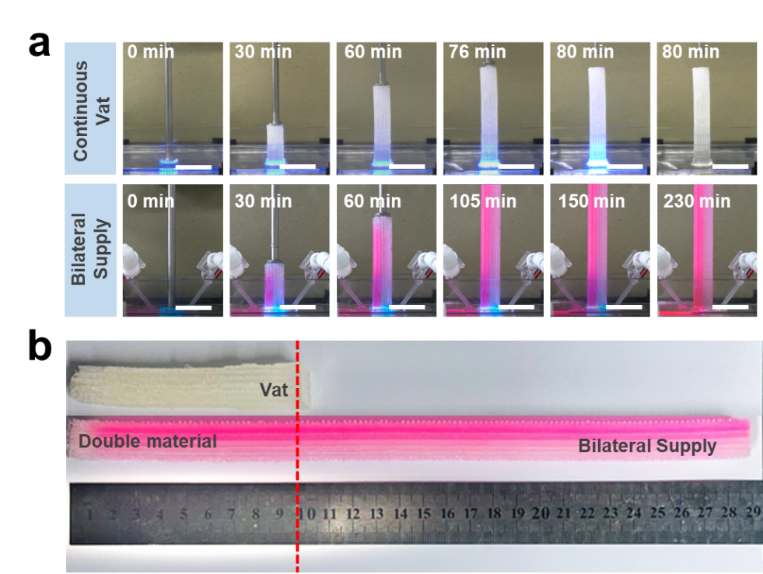
**

**Fig. S4.** Construction stability characterization along with time. (a) Optical captures of continuous vat polymerization of single materials, and bilateral extrusion construction two materials. (b) Optical images of corresponding printed results in (a). The stable construction time can be increased by over 3 times for multi-material continuous construction, with the stop of continuous construction due to the moving range limit of the moving platform rather than the sticking on the curing surface.


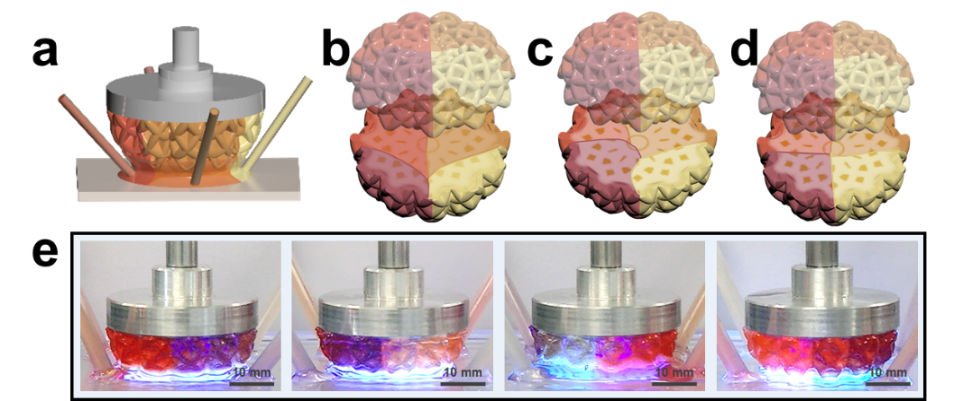


**Fig. S5.** Preparation of four-material porous pumpkin-shaped structure with controlled outer appearance and inner material distribution. (a) Scheme of the preparation configuration of the four-material porous pumpkin-shaped structure. (b) - (d) Schemes of the four-material pumpkin-shaped structure displaying the outer appearance of the four-material pumpkin-shaped structures with non-uniform (b, c) and uniform (d) inner distributions. (e) Optical captures of the construction process from different views, showing a clear interface between corresponding two adjacent materials.


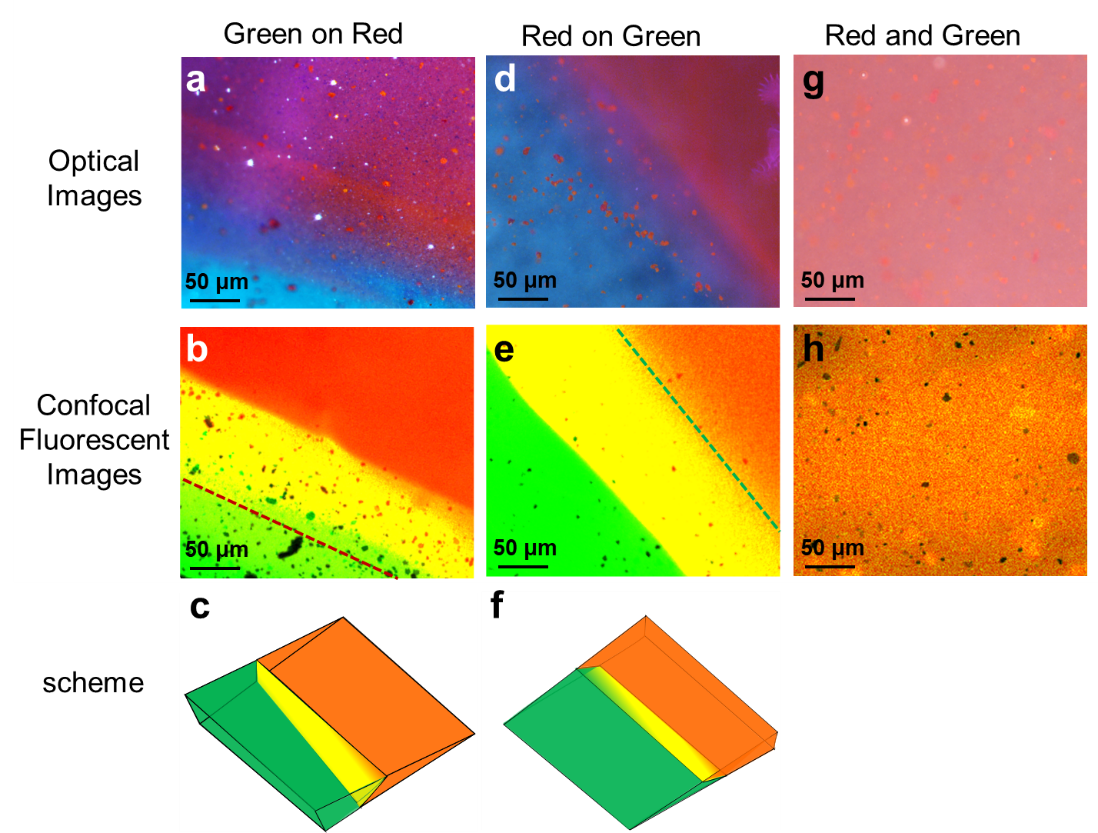


**Fig. S6.** Scheme and optical images of the structural physical overlap induced color change around the interface. (a) Optical image of the green structure stacking on red structure. (b) Confocal fluorescent image of the green structure stacking on red structure. (c) Scheme of the green structure stacking on red structure. (d) Optical image of the red structure stacking on green structure. (e) Confocal fluorescent image of red structure stacking on green structure. (f) Scheme of the red structure stacking on green structure. (g) Optical image of the structure uniformly loaded with red and green dyes. (h) Confocal fluorescent image of the structure uniformly loaded with red and green dyes. Generally, different stacking modes display different boundaries, where the structure on top shows clear boundary while the structure at the bottom shows blurry boundary, which are obvious in the confocal fluorescent images. While for the liquid resin uniformly loaded with red and green dyes, it displays different color both in the optical and confocal images comparing with the stacking structures. Therefore, the color mixing in Fig. 4M is due to the physical overlap of the structures, and is consistence with the green on red stacking, rather than fusion of the two materials before curing.


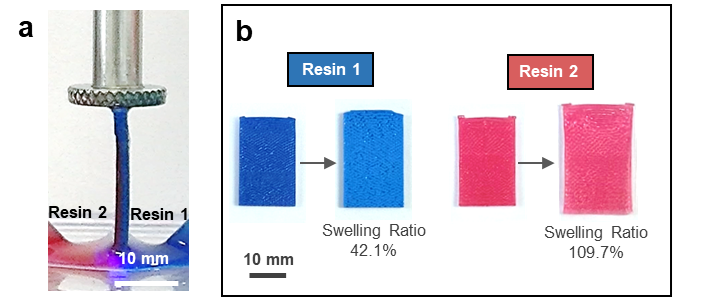


**Fig. S7.** (a) Optical capture of two-material laminated structure construction process (vertical construction speed, 20 μm/s). (b) Optical image of single material structure before and after swelling with anhydrous ethanol for 30 min. Resin 1 and 2 are commercial castable wax resin and commercial elastic resin dyed with red ink, respectively.

**Table S1.** Comparison of references concerning multi-material construction including different construction strategies.

|  | **Construction Strategy** | **DLP** | | | **SLA** | **DIW** | **Inkjet 3D Printing** |
| --- | --- | --- | --- | --- | --- | --- | --- |
|  | **Reference** | **Our Work** | **Ref. 1** | **Ref. 2** | **Ref. 3** | **Ref. 4** | **Ref. 5** |
|  |  |  | **Addit. Manuf. 2019, 27, 606-615** | **Nat. Commun. 2022, 13, 7931** | **J. Mater. Process. Technol. 2011, 211, 318-328** | **Nature 2019, 575, 330-335** | **Nature 2023, 623, 522-530** |
| **Single Material Construction** | **Construction Unit** | **Slice** | **Slice** | **Slice** | **Voxel** | **Voxel** | **Voxel** |
|  | **Construction format** | **Stacking of slices** | | | **Voxels to lines to slices, then stacking of slices** | | |
|  | **Construction resolution** | **Smallest ~ 15 μm (80 μm resolution light source)** | **5 μm Grid Lines (5 μm resolution light source)** | **Not mentioned, smallest 500 μm (25 μm resolution light source)** | **Not mentioned (0.102 mm beam source).** | **250 μm line width (200 μm Nozzle)** | **64 μm × 32 μm × 8 μm of one voxel** |
|  | **Maximum construction speed** | **150 μm/s along z axis** | **A few seconds of one layer (curing time + pumping time)** | **Not mentioned, at least 30s’ layer residual removal** | **Not mentioned (seconds’ settling time of each material)** | **Nozzle moving speed of 20 mm/s along x-y plane** | **16 mm/h (~4.5 μm/s) along z axis** |
|  | **Maximum Construction Area** | **15 cm × 8 cm without cooling** | **A few millimeters’ scale** | **18 cm × 13 cm** | **/** | **Not mentioned (~ 60 cm × 60 cm maximum)** | **50 cm × 24.5 cm** |
|  | **Maximum Construction Height** | **~29 cm (moving platform upper limit)** | **A few millimeters’ scale** | **/** | **12.7 cm** | **/** | **20 cm** |
|  | **Material Utilization Efficiency** | **On demand** | **Vat Residual** | **Vat Residual** | **Vat Residual** | **On demand** | **On demand** |
| **Multi-material Construction** | **Simultaneous Construction Number of Materials** | **4 or more** | **1** | **1** | **1** | **1 (multiple nozzles, not independently switchable)** | **4 (four independent printheads)** |
|  | **Multi-Material Switching Continuity** | **Continuous** | **Discontinuous** | **Discontinuous** | **Discontinuous** | **Continuous** | **Continuous** |
|  | **Interfacial Resolution** | **Smallest of ~15 μm** | **/** | **~ 100 μm** | **/** | **~ 200 μm** | **/** |
|  | **Interfacial Mechanical** | **Interfacial bonding > Single material** | **/** | **Interfacial bonding > Single material** | **/** | **Interfacial bonding > Single material** | **/** |

**Table S2.** Extrusion parameters for construction two-material zigzag interfaces in Fig. 2B.

|  | **Zigzag Interface** | **Ⅰ** | **Ⅱ** | **Ⅲ** | **Ⅳ** | **Ⅴ** | **Ⅵ** | **Ⅶ** | **Ⅷ** | |
| --- | --- | --- | --- | --- | --- | --- | --- | --- | --- | --- |
| **Blue Resin** | **Initial extrusion rate of**  **one period (μl/min)** | 15 | 30 | 30 | 20 | 30 | 20 | 30 | 20 | |
|  | **Turning extrusion rate of one period (μl/min)** | **/** | **/** | 0 | 10 | 0 | 10 | 0 | 10 | |
|  | **Finish extrusion rate of**  **one period (μl/min)** | 15 | 0 | 30 | 20 | 30 | 20 | 30 | 20 | |
|  | **Period (min)** | 30 | 30 | 20 | 20 | 10 | 10 | 5 | 5 | |
|  | **Repeat times of period** | 1 | 1 | 1.5 | 1.5 | 3 | 3 | 6 | 6 | |
| **Origin Resin** | **Initial extrusion rate of**  **one period (μl/min)** | 15 | 0 | 0 | 10 | 0 | 10 | 0 | 20 | |
|  | **Turning extrusion rate of one period (μl/min)** | **/** | **/** | 30 | 20 | 30 | 20 | 30 | 10 | |
|  | **Finish extrusion rate of**  **one period (μl/min)** | 15 | 30 | 0 | 10 | 0 | 10 | 0 | 20 | |
|  | **Period (min)** | 30 | 30 | 20 | 20 | 10 | 10 | 5 | 5 | |
|  | **Repeat times of period** | 1 | 1 | 1.5 | 1.5 | 3 | 3 | 6 | 6 | |
| **Morphology** | | 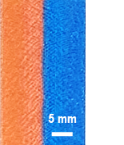 | 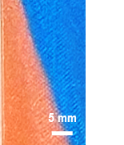 | 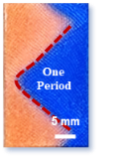 | 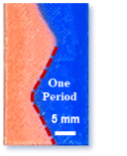 | 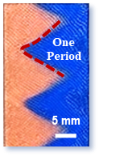 | 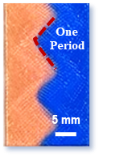 | 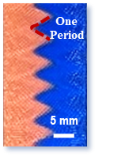 | 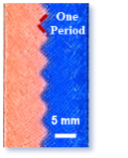 |  |

Note: Red dotted lines in the “Morphology” row “III-VIII” columns indicate the interface that can be printed through one period of liquid extrusion.**Table S3.** Morphology characterization of interfaces printed from two-material continuous liquid extrusion with different resin viscosities.

| Liquid Resins | Elastic / Clear | Grey / Elastic | Elastic / Wax | Clear / Wax | Grey / Clear | Grey / Wax |
| --- | --- | --- | --- | --- | --- | --- |
| Liquid Extrusion Rate | 27 μL/min /  15 μL/min | 15 μL/min /  22 μL/min | 20 μL/min /  15 μL/min | 15 μL/min /  20 μL/min | 18 μL/min /  15 μL/min | 15 μL/min /  16 μL/min |
| Interface Morphology | 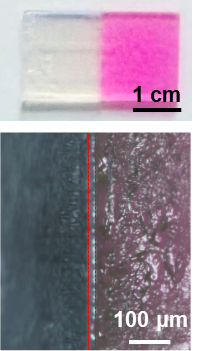 | 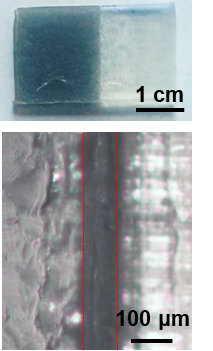 | 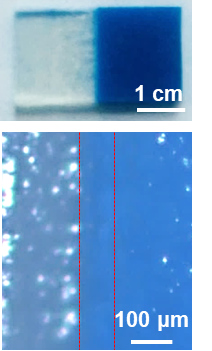 | 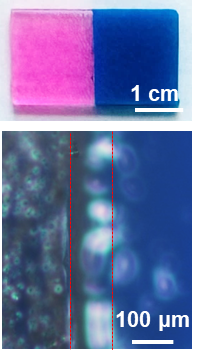 | 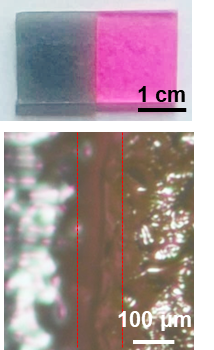 | 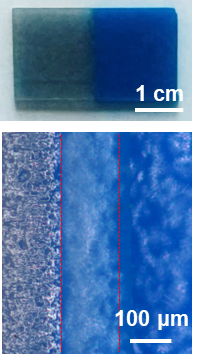 |
| Fusion Width | ~ 15 μm | ~ 80 μm | ~ 90 μm | ~ 100 μm | ~ 105 μm | ~ 140 μm |

The determination of the fusion zone is based on the surface topography and the transparency of the printed structure[*49*].

**Movie S1.**

Construction process of bi-material lamellar structure curved interface.

**Movie S2.**

Tensile test along the interface direction for the samples prepared by bilateral extrusion construction and replication approach.

**Movie S3.**

Construction process of four-material porous pumpkin-shaped structure.

**Movie S4.**

Real time monitoring of the 3D material distribution defined trajectories of magnetic-responsive fishes.
